# Multi-omics analysis reveals the associations between altered gut microbiota, metabolites, and cytokines during pregnancy

**DOI:** 10.1128/msystems.01252-23

**Published:** 2024-02-07

**Authors:** Ting Huang, Xinyuan Liang, Han Bao, Guangyu Ma, Xiaomei Tang, Huijuan Luo, Xiaomin Xiao

**Affiliations:** 1 Department of Obstetrics and Gynecology, The First Affiliated Hospital of Jinan University, Guangzhou, China; 2 Department of Obstetrics, The Second Clinical Medical College, Jinan University (Shenzhen People’s Hospital), Shenzhen, China; University of Massachusetts Medical School, Worcester, Massachusetts, USA

**Keywords:** pregnancy, gut microbiota, metabolism, cytokines, 16S rRNA

## Abstract

For embryo implantation and fetal development, the maternal immune system undergoes dramatic changes. The mechanisms involved in inducing alterations of maternal immunity have not been fully clarified. Gut microbiome and metabolites were thought to influence the host immune response. During normal pregnancy, notable changes occur in the gut microbiota and metabolites. However, the relationship of these alterations to immune function during pregnancy remains unclear. In this study, we examined gut microbiota, fecal metabolites, plasma metabolites, and cytokines in pregnant women and non-pregnant women. Our findings revealed that, in comparison to non-pregnant women, pregnant women exhibit a significant increase in the relative abundance of *Actinobacteriota* and notable differences in metabolic pathways related to bile acid secretion. Furthermore, there was a marked reduction in pro-inflammatory cytokines levels in pregnant women. Correlation analyses indicated that these alterations in cytokines may be linked to specific gut bacteria and metabolites. Bacteria within the same microbial modules exhibited consistent effects on cytokines, suggesting that gut bacteria may function as functional groups. Mediation analysis further identified that certain bacteria might influence cytokines through metabolites, such as bile acids and arachidonic acid. Our findings propose potential biological connections between bacteria, metabolites, and immunity, which require further validation in future studies.

**IMPORTANCE:**

A great number of studies have focused on diseases induced by intestinal microecological disorders and immune imbalances. However, the understanding of how intestinal microbiota interacts with immunity during normal pregnancy, which is fundamental to studying pathological pregnancies related to intestinal microbiota disturbances, has not been well elucidated. Our study employed multi-omics analysis to discover that changes in gut microbiota and metabolites during pregnancy can impact immune function. In addition, we identified several metabolites that may mediate the effect of gut microbes on plasma cytokines. Our study offered new insights into our understanding of the connections between the gut microbiome, metabolome, and the immune system during pregnancy.

## INTRODUCTION

Pregnancy represents a distinct physiological state characterized by fluctuations in hormone levels, alterations in body structure, and modifications in immune status. These changes play a pivotal role in facilitating embryo implantation and fetal growth (1). During the first and early second trimesters of pregnancy, the maternal immune system tends to exhibit a pro-inflammatory profile, which transitions to an anti-inflammatory state in the subsequent phase (1). As labor approaches, there is a shift back to a pro-inflammatory state in maternal immunity, which serves to promote labor (1). However, the precise mechanisms governing these dynamic immunological changes during pregnancy remain incompletely elucidated. Factors such as the presence of the fetal allograft, fluctuations in hormone levels, and the influence of placental cytokines have been implicated in modulating the immune response (2, 3). Recent findings from an animal experiment have shed light on the role of the microbiome in pregnancy-related immune regulation and outcomes (4). This study compared the immune adaptations and pregnancy outcomes of germ-free mice and conventional mice following pregnancy. Notably, despite having a similar total number of fetuses, germ-free mice exhibited a markedly lower count of living fetuses compared to their conventional mice (4). These results highlight the potential significance of the microbiome in shaping both immune responses during pregnancy and pregnancy outcomes.

The gut microbiome is the largest microbial community in the human body (5, 6). It plays a key role in the regulation of host functions, such as fermentation of dietary fibers (7), pathogen offense (8), metabolic function (9), and immune maturation (10). The gut microbial community in early pregnancy was similar to that of non-pregnant women. In late pregnancy, the abundance of *Firmicutes* decreased, while the abundance of *Proteobacteria* and *Actinobacteria* increased (11). Significant evidence has demonstrated that disturbed gut microbiota promotes preeclampsia by affecting autophagy and M2 polarization of macrophages (12). This study underscores the significance of the gut microbiome in modulating the immune response during pregnancy. However, the precise mechanisms underlying the interaction between the altered gut microbiota in normal pregnant women and host immunity remain elusive, necessitating further research. A deeper understanding of the associations between the gut microbiome and pregnancy immunity may provide new ideas for the treatment of pathological pregnancy.

The microbiome can account for only approximately 10% of the variability in cytokine levels (13). The mutual immune regulation between the microbiome and the host is primarily mediated through metabolites, rather than direct interactions between specific microbes and immune cells (13). A recent study reported that numerous plasma metabolites undergo pronounced shifts during pregnancy (14). The question of whether and how these metabolites, which undergo significant changes during pregnancy, interact with immune cells remains a subject that requires further investigation.

In this study, we analyzed the gut microbiome, fecal metabolites, plasma metabolites, and cytokines of pregnant women, and compared them with those of healthy non-pregnant women. We used the integrated method to reveal the associations among maternal microbiome, metabolites, and cytokines. This study expands our current knowledge regarding how altered gut microbiota and metabolites interact with the host during pregnancy.

## RESULTS

### Clinical characteristics of participants

This study recruited 30 healthy pregnant women and 15 healthy non-pregnant women as controls. The average age of the pregnant participants was 26.67 ± 2.15 years, compared to 26.40 ± 1.84 years for the control group. Furthermore, the mean pre-pregnancy body mass index (BMI) for the pregnancy group stood at 19.90 ± 1.46 kg/m^2^, while it was 19.57 ± 0.67 kg/m^2^ for the controls. There were no statistically significant differences in age (*P* = 0.684) and BMI (*P* = 0.312) between the two groups (Table S1).

### Variation of plasma cytokines in pregnant women

**Fig 1:**
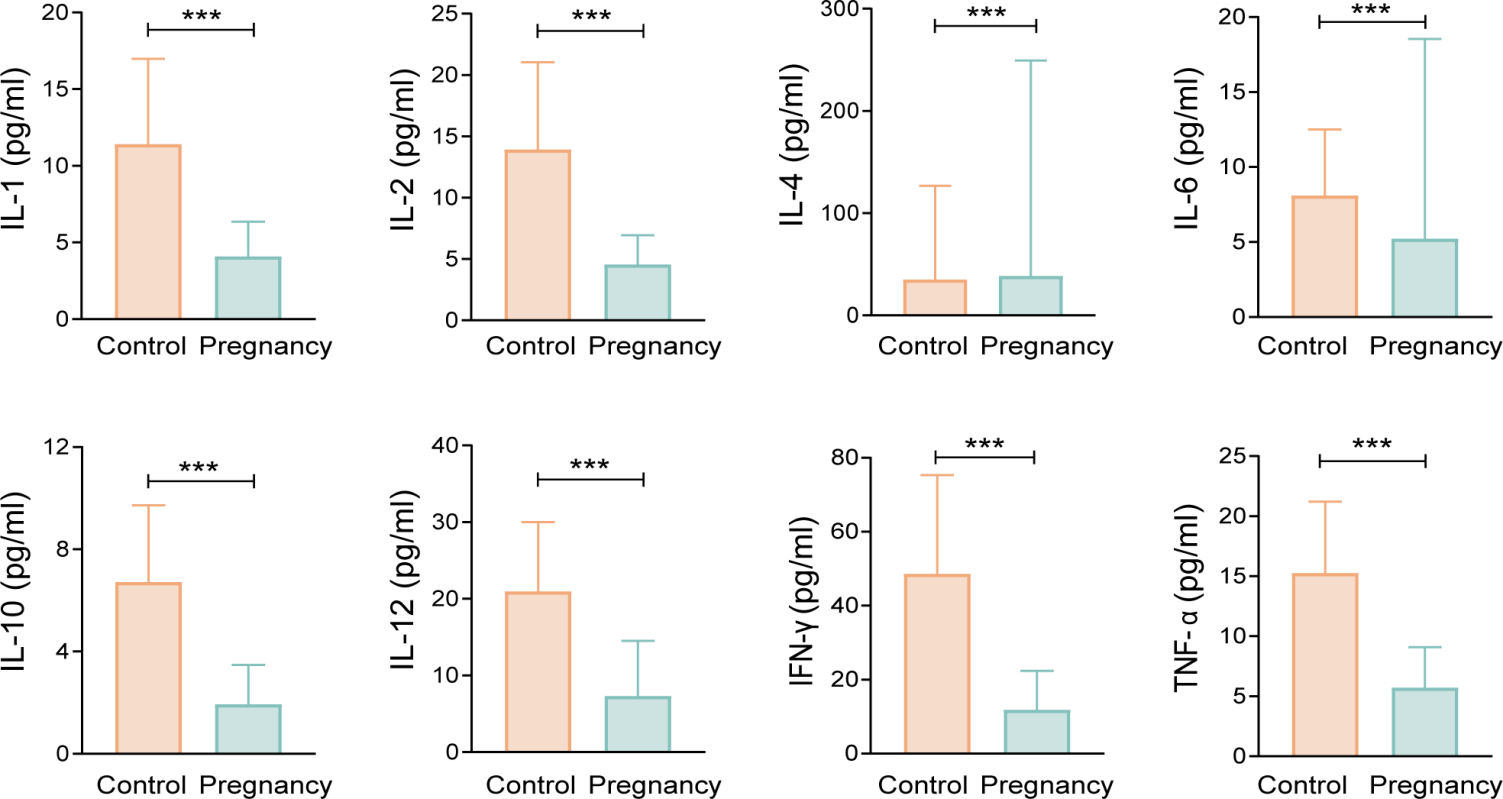
Comparison of plasma cytokines between the pregnancy group and control group. Differences between groups were calculated using the Wilcoxon rank-sum test. ***P < 0.001.

The immune profiles of pregnant and non-pregnant women exhibited notable differences. Pregnant women displayed reduced levels of pro-inflammatory cytokines, including interleukins IL-1β, IL-2, IL-6, IL-12, interferon gamma (IFN-γ), and tumor necrosis factor alpha (TNF-α), while exhibiting elevated levels of the anti-inflammatory cytokine IL-4 (Fig. 1). Conversely, IL-10, another anti-inflammatory cytokine, increased in the control group (Fig. 1). This finding suggested that the immune state during pregnancy is characterized by a relative immunosuppression compared to the immunological status observed in the non-pregnant condition.

### The relationship between gut microbiota and plasma cytokines

**Fig 2:**
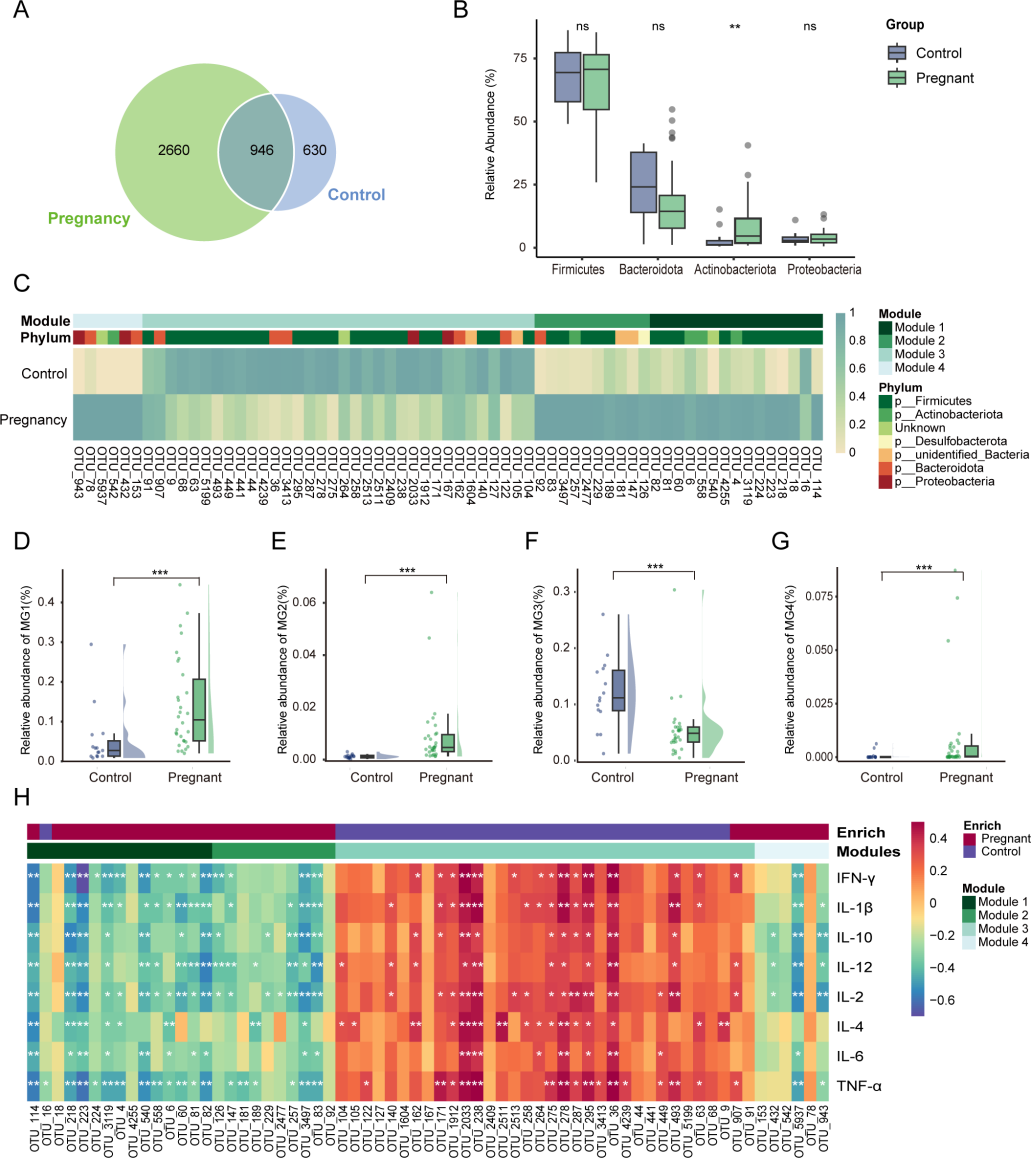
Characteristics of gut microbial community in pregnancy group and control group. (**A**) Venn diagram indicates the number of unique and shared OTUs between the pregnancy and control groups. (**B**) Comparison of the relative abundance of the top four phyla in the gut microbiota of the pregnancy and control groups. (**C**) A heatmap illustrating that the relative abundance of microbial biomarkers varied in pregnant women and controls. (**D–G**) Comparison of the relative abundance of modules 1–4 between the pregnancy and control groups. (**H**) Heatmap of Spearman correlation analysis between gut microbiota and plasma cytokines. The colors on the heatmap represent the magnitude of the correlation coefficients, with orange/red indicating a positive correlation and green/blue indicating a negative correlation. *P < 0.05, **P < 0.01, and ***P < 0.001.

Previous studies have reported distinct gut microbiota between pregnant women and non-pregnant women (11, 15, 16). Therefore, we compared the gut microbiota between the pregnancy group and the control group. We observed a total of 2,660 unique operational taxonomic units (OTUs) in the pregnancy group. In contrast, the control group exhibited a significantly lower count with only 630 OTUs identified (Fig. 2A). Furthermore, *Firmicutes* dominated as the most prevalent phylum in the gut microbiota of both the pregnancy group and the control group (65.28% vs 67.82%, *P* = 0.80) (Fig. 2B). The relative abundance of *Bacteroidota* was lower in the pregnancy group compared to the control group (18.61% vs 24.09%, *P* = 0.23) (Fig. 2B). Conversely, the pregnancy group exhibited a higher relative abundance of *Actinobacteriota* and *Proteobacteria* compared to the control group (*Actinobacteriota*: 9.15% vs 2.98%, *P* = 0.002; *Proteobacteria*: 4.04% vs 3.48%, *P* = 0.76) (Fig. 2B), which is broadly consistent with the findings of Koren et al. (11).

Given OTU was the basic unit of taxonomy, we established a random forest model based on OTU level to identify the microbial biomarkers that can distinguish between these two peoples (Fig. S1; Table S2). Microbes may survive and adapt as guilds to respond to environmental perturbations. In microbial ecology research, it is common to preliminarily identify functional microbial communities by partitioning microbial modules or co-abundance groups (17–19). Therefore, we selected the top 100 OTUs with the highest contribution to distinguish the two groups in random forest model and clustered them into four modules by using WGCNA methods (Fig. 2C; Table S3). In the pregnant group, the abundance of modules 1, 2, and 4 was significantly higher than that of the control group, while module 3 exhibited a significantly lower abundance compared to the control group (Fig. 2D through G). Furthermore, we conducted Spearman correlation analysis to investigate their relationships with cytokines. As illustrated in Fig. 2H, most of the OTUs enriched in modules 1, 2, and 4 in the pregnant group exhibited a negative correlation with pro-inflammatory cytokines such as IL-2 and TNF-α, while most of the OTUs in module 3, depleted in the pregnant women, showed a positive correlation with pro-inflammatory cytokines. This suggests that microbes within the same module may have a broadly consistent impact on the immune system. Specifically, OTU_4 and OTU_6, belonging to *Bifidobacterium*, displayed significantly higher abundance in the gut of pregnant women compared to the control group (Fig. S2), and they exhibited a negative correlation with TNF-α, IFN-γ, and IL-1β. Similarly, OTU_16 and OTU_82, belonging to the *Ruminococcus* genus, showed significantly higher abundance in pregnant women (Fig. S2). OTU_16 was negatively correlated with TNF-α, and OTU_82 exhibited negative correlations with cytokines such as TNF-α, IFN-γ, and IL-1β. *Bifidobacterium* and *Ruminococcus* are recognized as anti-inflammatory bacteria known for producing short-chain fatty acids (20, 21). This implied that the increased abundances of certain anti-inflammatory bacteria during pregnancy may be linked to a reduction in pro-inflammatory cytokines levels.

### Alterations in the fecal and plasma metabolites in pregnant women and their relationships with cytokines

**Fig 3:**
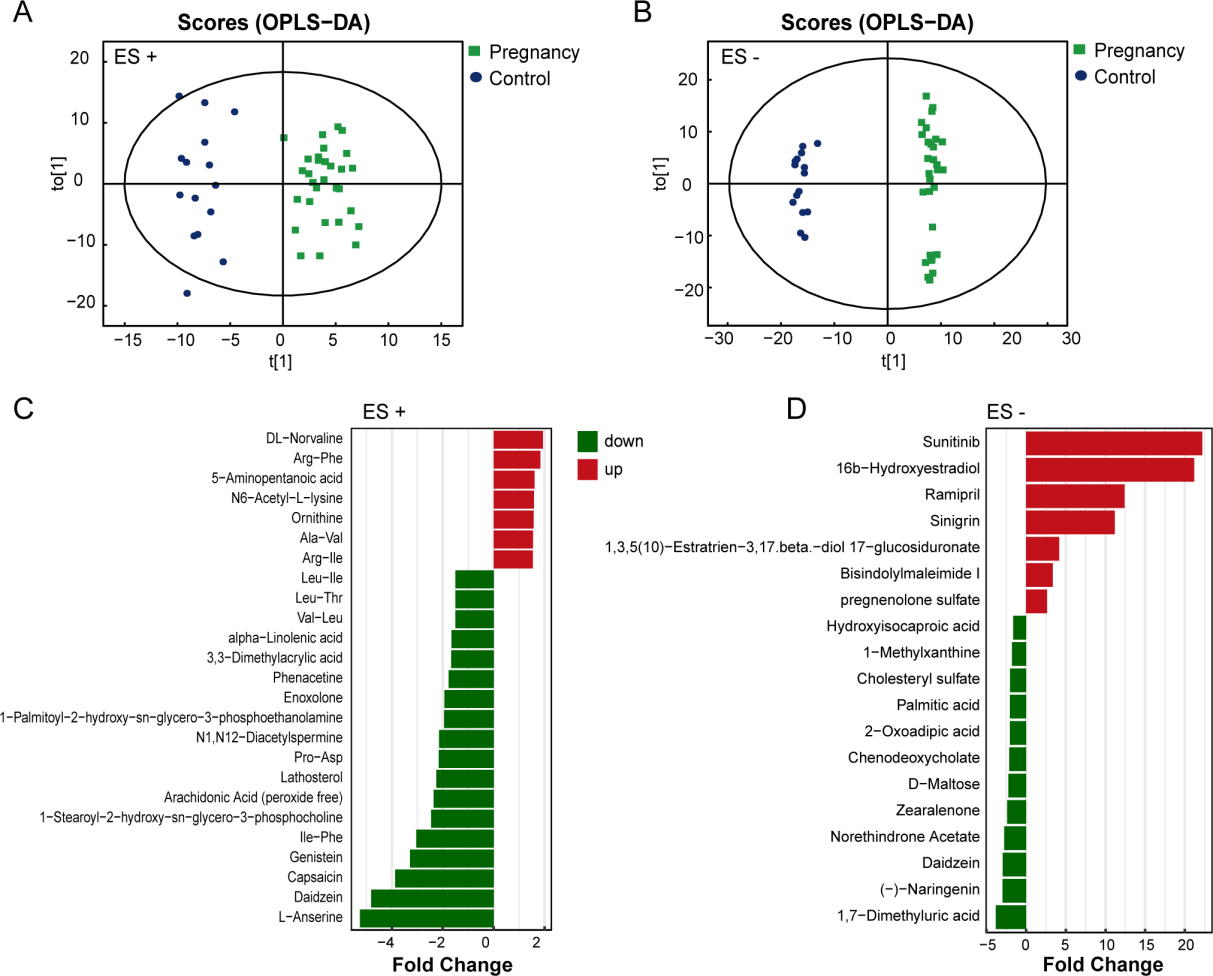
Fecal metabolite profiles of the pregnancy and control group. (**A and B**) OPLS-DA score plot of fecal samples from pregnancy group and control group in ES+ and ES−, respectively. Samples from the same group are represented by points of the same color. The distribution of points reflects differences between and within groups. (**C and D**) Differential fecal metabolites in ES+ and ES− between pregnant and non-pregnant women. The *x*-axis represents the values of fold change in differential expression between pregnancy group samples and control group samples. Red color represents fold change >1, indicating upregulated differential metabolites in the pregnancy group, while green color represents fold change <1, indicating downregulated differential metabolites in the pregnancy group.

**Fig 4:**
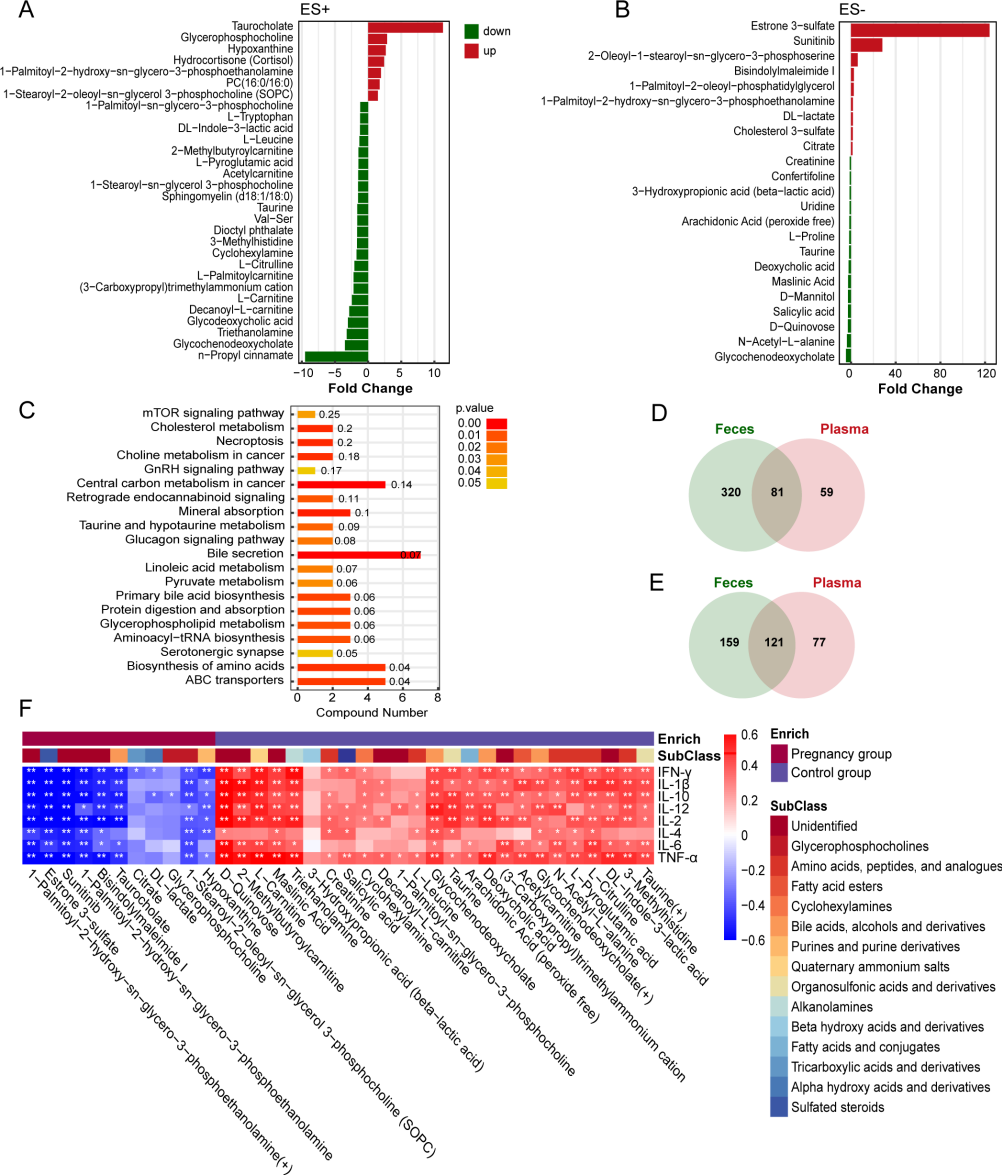
Plasma metabolites profiles of the pregnancy and control group. (**A and B**) Differential plasma metabolites in ES+ and ES− between pregnant and non-pregnant women. The *x*-axis represents the values of fold change in differential expression between pregnancy group samples and control group samples. Red color represents fold change >1, indicating upregulated differential metabolites in the pregnancy group, while green color represents fold change <1, indicating downregulated differential metabolites in the pregnancy group. (**C**) KEGG pathway enrichment analysis of differentially expressed plasma metabolites. The *y*-axis represents various KEGG metabolic pathways, while the *x*-axis represents the number of differentially expressed metabolites within each KEGG pathway. Colors denote the *P* values from the enrichment analysis, with darker colors indicating smaller *P* values and greater enrichment significance. (**D**) Venn diagram of fecal metabolites and plasma metabolites in ES+. (**E**) Venn diagram of fecal metabolites and plasma metabolites in ES−. (**F**) Heatmap of correlations between metabolites and cytokines. The colors on the heatmap represent the magnitude of the correlation coefficients, with red indicating a positive correlation and blue indicating a negative correlation. *P < 0.05, **P < 0.01, and ***P < 0.001.

Metabolomics could reflect biochemical process alterations of host influenced by genetics, environment, and microbiome (22). Therefore, we employed untargeted liquid chromatography-mass spectrometry (LC-MS)-based metabolomics to analyze fecal and plasma samples in order to investigate metabolic changes during pregnancy. In the fecal samples, a total of 401 metabolites were identified in positive ion mode (ES+), while 280 metabolites were identified in negative ion mode (ES−). In the plasma samples, 140 metabolites at ES+ and 198 metabolites at ES− were identified. The overall metabolic variations between pregnant women and non-pregnant women were further revealed through orthogonal partial least-squares discriminant analysis (OPLS-DA). A distinct separation of metabolome between the two groups was observed both in ES+ and ES− (Fig. 3A and B; Fig. S3), indicating substantial metabolic alterations in feces and plasma during pregnancy. The permutation test was used to validate the robustness of OPLS-DA model. With the gradual decrease of replacement retention, both *R*^2^ and *Q*^2^ of the random model decreased gradually, suggesting no overfitting issue arise, and the model was robust (Fig. S4A through D). To identify significantly differential metabolites, we calculated variable importance in the projection (VIP) scores from the OPLS-DA models. Metabolites, whose VIP score exceeded 1 and *P* value was <0.05, were regarded as differential metabolites between groups. In summary, a total of 44 differential fecal metabolites and 53 differential plasma metabolites were detected in pregnant women compared to non-pregnant women (Fig. 3C and D; Fig. 4A and B). The majority of differential fecal metabolites are lipids and lipid-like molecules, such as arachidonic acid and chenodeoxycholate, both of which were downregulated in the pregnant group (Fig. 3C and D). In plasma metabolites, a similar downregulation of arachidonic acid was observed (Fig. 4B). Additionally, substances related to bile acid metabolism were decreased in pregnant females, including glycochenodeoxycholate, glycodeoxycholic acid, and deoxycholic acid (Fig. 4A and B). The Kyoto Encyclopedia of Genes and Genomes (KEGG) enrichment pathway further revealed that bile acid secretion is the most significantly altered pathway, involving the highest number of metabolites (Fig. 4C). Arachidonic acid and bile acids have both been reported to be associated with inflammation (23–25). They may be potential factors influencing immune responses during pregnancy.

In the process of digestion, food undergoes a complex series of biochemical reactions and produces various metabolites. The gut microbiota plays a role in metabolizing specific nutrients and regulating the levels of intestinal metabolites. Metabolites originating from dietary sources and those produced by the gut microbiota can enter the blood circulation through the enterohepatic circulation, exerting biological effects. As a result, we intersected fecal metabolites with plasma metabolites, identifying 81 common metabolites in ES+ and 121 common metabolites in ES− (Fig. 4D and E). Among the 202 common metabolites, 36 exhibited intergroup differences in plasma, forming the focus of our further investigations (Fig. S5). We further evaluated the correlations between these shared metabolites and cytokines. Our findings revealed that certain enriched metabolites in pregnant women were negatively correlated with pro-inflammatory cytokines, while depleted metabolites in pregnant women were positively correlated with pro-inflammatory cytokines (Fig. 4F). For instance, we observed a notable negative correlation between taurocholate, which is enriched in the pregnant group, and pro-inflammatory cytokines such as IFN-γ, IL-1β, and TNF-α. Conversely, metabolites like glycochenodeoxycholate, deoxycholic acid, and arachidonic acid, which are enriched in the control group, exhibited a positive correlation with several pro-inflammatory cytokines.

### Association of gut microbiota, metabolites, and cytokines

**Fig 5:**
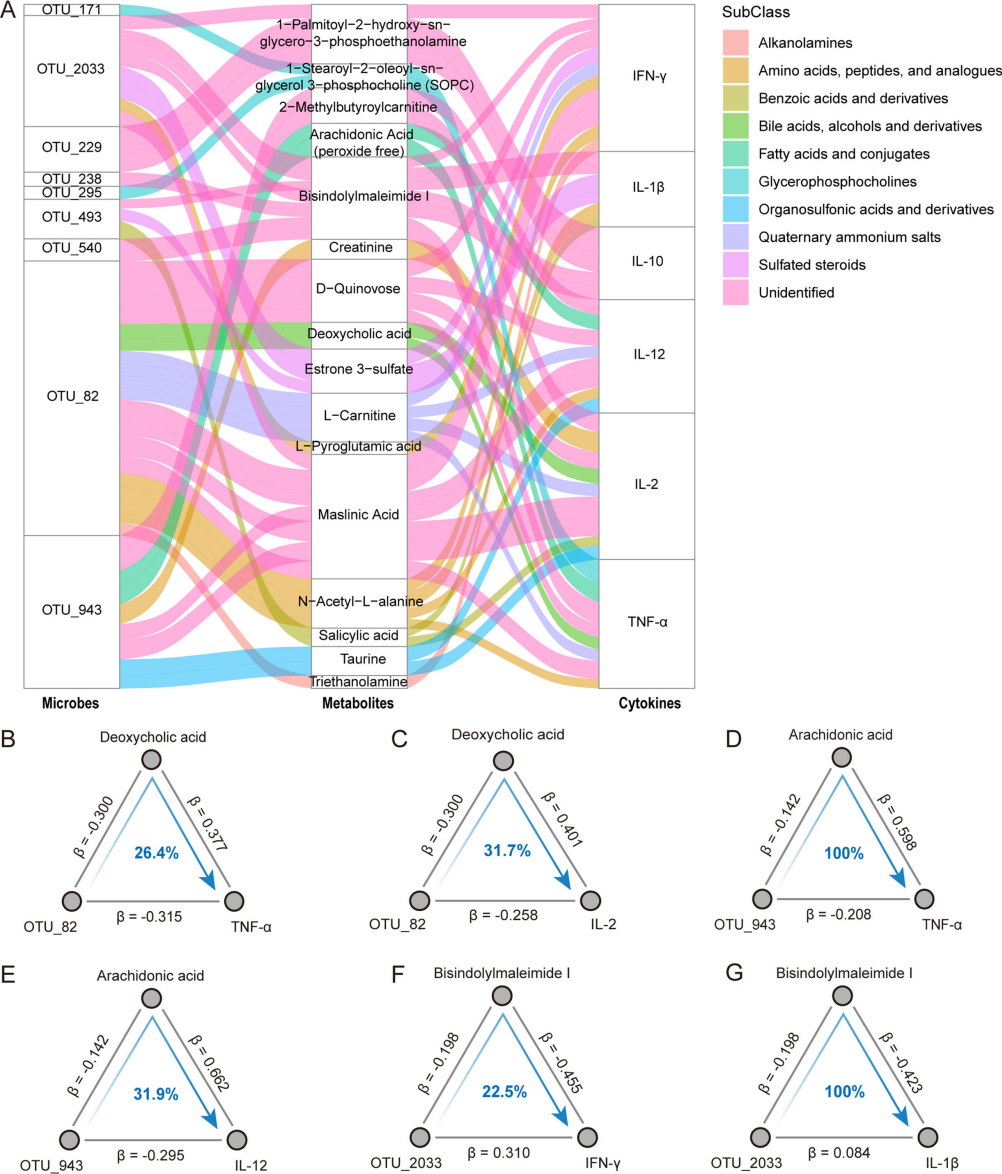
Mediation linkages between gut microbiota, metabolites, and cytokines. (**A**) The Sankey diagram displays mediation linkages among gut microbiota, metabolites, and cytokines. Columns from left to right show the OTUs, metabolites, and cytokines, respectively. The curved lines across the columns indicate the mediation effects. Different colored curves represent the subclasses to which the metabolites belong. (**B**) Analysis of the effect of OTU_82 on TNF-α as mediated by deoxycholic acid [indirect effect: −0.113, 95% boot confidence interval (CI): −0.298, −0.038]. The proportion of the mediation effect is labeled at the center of the ternary diagrams. (**C**) Analysis of the effect of OTU_82 on IL-2 as mediated by deoxycholic acid (indirect effect: −0.120, 95% boot CI: −0.324, −0.025). (**D**) Analysis of the effect of OTU_943 on TNF-α as mediated by arachidonic acid (indirect effect: −0.085, 95% boot CI: −0.220, −0.008). (**E**) Analysis of the effect of OTU_943 on IL-12 as mediated by arachidonic acid (indirect effect: −0.094, 95% boot CI: −0.241, −0.008). (**F**) Analysis of the effect of OTU_2033 on IFN-γ as mediated by bisindolylmaleimide I. (**G**) Analysis of the effect of OTU_2033 on IL-1β as mediated by bisindolylmaleimide I.

The gut microbiota is considered to play a key role in the host metabolism, capable of both regulating the host’s metabolites and producing its own metabolites which have a significant impact on the host (26). In this study, cytokines were associated with both gut microbiota and their metabolites. Moreover, the correlations between cytokines and metabolites were more numerous and stronger than those between cytokines and microbiota. Several studies have reported on the mediating role of metabolites in the interaction between microbiota and immune functions or disease phenotypes (19, 27, 28). Therefore, we hypothesize that metabolites may mediate the association between gut microbiota and cytokines during pregnancy. We performed directional mediation analysis to further explore which metabolites may mediate the impact of gut microbiome on immunity. Finally, a total of 46 linkages among microbes, metabolites, and cytokines were identified (Fig. 5A; Table S4). We found that some microbes may alleviate the systemic inflammatory response by inhibiting pro-inflammatory metabolites. For instance, OTU_82, enriched in pregnant women and belongs to *Ruminococcus callidus*, decreased the TNF-α and IL-2 levels by reducing deoxycholic acid levels (Fig. 5B and C; Fig. S6A and B). Moreover, OTU_943, a bacteria enriched in the pregnancy group, contributes to lower TNF-α and IL-12 levels by decreasing arachidonic acid (Fig. 5D and E; Fig. S6C and D). Conversely, several metabolites mediate the impact of gut microbiota on pro-inflammatory cytokines. OTU_2033 depleted in pregnant women, which may increase the IFN-γ and IL-1β by decreasing the bisindolylmaleimide I level (Fig. 5F and G). Taken together, our results suggest that gut microbes may alter plasma cytokines by interacting with host metabolites.

## DISCUSSION

To investigate the interaction between host and microbiota during pregnancy and offer preliminary insights into this aspect, we explored the associations among gut microbiota, metabolites, and plasma cytokines of pregnant women and non-pregnant women. We observed alterations in the gut microbiome, fecal metabolite, and plasma metabolite characteristics in pregnant women. Further analysis indicated a correlation between these changes and variations in cytokine levels. Mediation analysis revealed the potential role of metabolites in mediating the connection between gut microbiota and cytokines. To the best of our knowledge, the associations among altered gut microbiota, metabolites, and plasma cytokines during normal pregnancy were first explored in this study.

To ensure the normal progression of pregnancy, the maternal immune system shifts toward a Th2-type response in the mid to late stages of gestation, exhibiting more anti-inflammatory characteristics. Th1 cells secrete pro-inflammatory cytokines, such as IL-2, IFN-γ, and TNF-α (29), and are involved in specific autoimmune responses and cellular immunity. Th2 cells, which produce IL-4 and IL-10, facilitate the proliferation of B cells, antibody production, and tolerance to allogeneic rejection (30). In our study, pro-inflammatory cytokines such as IL-1β, IL-6, IFN-γ, and TNF-α exhibited a downregulation in the plasma of pregnant women, while the anti-inflammatory cytokine IL-4 showed an upregulation, largely consistent with the result of a previous study (31). However, the IL-10 levels in pregnant women were found to be lower than those in non-pregnant women, contradicting the findings of prior research (31, 32). This discrepancy may be due to the inconsistency between the characteristics of cytokines in whole blood and those isolated *in vitro*. Furthermore, we did not observe a marked increase in anti-inflammatory cytokines in the late stages of pregnancy. One possible explanation for this observation is that our control group consisted of non-pregnant women. Compared to them, pregnant women typically exhibit a state of relative immunosuppression throughout their pregnancy, a conclusion that is supported by the work of Abu-Raya and colleagues (33). Additionally, the timing of blood sample collection from the pregnant participants was prior to the onset of labor. During this phase, the fetus remains in a rapid growth and development stage, with the maternal local immune response predominantly skewed toward Th2-type dominance.

Gut microbiota is closely related to the host immune system, which may directly or indirectly influence mucosal immunity and ultimately affect systemic immune function (13, 34). To explore immune alteration during pregnancy whether affected by the intestinal microbiota, we analyzed the characteristics of the gut microbiota and immune status in pregnant women and non-pregnant women and conducted a correlation analysis between these factors. Compared to non-pregnant women, pregnant women showed an increased abundance of *Actinobacteriota* and *Proteobacteria*, which is largely consistent with the findings of Koren et al. (11). Gut microbiota does not exist in isolation; they work as guilds, collectively thriving or declining in response to environmental changes (35, 36). In ecological and biological research, identifying microbial modules helps researchers understand how complex microbial ecosystems operate. Therefore, we categorized the gut microbiota into clusters, dividing them into multiple modules to reduce the dimensionality of the microbial data. In the pregnant group, modules 1, 2, and 4 demonstrated significantly higher abundance and were negatively correlated with certain pro-inflammatory cytokines. This seems to indicate that microbial members belonging to the same module have the same or similar functions (37). Additionally, *Bifidobacterium* are known as probiotics in the human intestine. Some members of *Bifidobacterium* have been shown to exert an immunological effect (38, 39). They induce high levels of anti-inflammatory cytokines or reduce pro-inflammatory cytokines through pili (40), extracellular polysaccharides (41, 42), etc. OTUs belonging to *Bifidobacterium* demonstrated a negative correlation with pro-inflammatory cytokines, indicating a relationship between *Bifidobacterium* and the reduction of pro-inflammatory cytokines during pregnancy.

Metabolites play roles in the immune system that are independent of their conventional functions as sources or intermediates in biosynthesis and bioenergetics (43). Specific metabolites have the capacity to either regulate cytokine production or demonstrate cytokine-like effects (43). Hence, we analyzed the metabolic characteristics of pregnant women and the associations between differential metabolites and cytokines. The most significant metabolic pathway that differed between pregnant and non-pregnant women was the bile acid secretion pathway, which also involved the largest number of differential metabolites. The metabolism of bile acids is a complex process. Primary bile acids such as cholic acid and taurocholic acid are synthesized in the liver and secreted into the bile, subsequently entering the intestine (44). Enzymes produced by intestinal microbiota further metabolize primary bile acids into secondary bile acids (45), including deoxycholic acid and glycodeoxycholic acid. Subsequently, approximately 95% of bile acids in the intestine are reabsorbed into the portal vein before reaching the distal ileum, completing one cycle of enterohepatic circulation (46). In our study, primary bile acids such as taurocholate in the plasma of pregnant women were upregulated, while secondary bile acids such as deoxycholic acid, glycochenodeoxycholate, and glycodeoxycholic acid were downregulated. This may be attributed to reduced farnesoid X receptor (FXR) activity in the intestines during pregnancy (47). FXR can enhance the expression of organic solute transporter α and β, promoting the reabsorption of bile acids into the portal vein (48). Consequently, the levels of secondary bile acids in the plasma during pregnancy were reduced. Studies have reported that serum levels of conjugated bile acids, especially taurine-conjugated ones, dominate the bile acid profile in late pregnancy (49). As gestational weeks increase, taurocholic acid levels rise, while deoxycholic acid levels decrease (49), consistent with our research findings. Bile acids play an essential role in regulating immunity (50, 51). Recently, bile acids have been reported to influence host immunity by modulating adaptive immune cells (52). In the present study, we observed negative associations between taurocholate, which was significantly increased in the pregnant group, with pro-inflammatory cytokines such as TNF-α and IL-1β. A fundamental research confirmed that high concentrations of taurocholic acid can inhibit the production and gene expression of TNF-α and IL-1β (53). Deoxycholic acid and glycochenodeoxycholate, which were depleted in pregnant women, were positively correlated with pro-inflammatory cytokines. Glycochenodeoxycholate and deoxycholic acid have both been demonstrated to possess inflammatory activation properties (24, 25). Our findings indicated a strong correlation between bile acid metabolism and the variations in plasma cytokine levels during pregnancy. Furthermore, a significant portion of metabolites present in the blood originates from the intestinal tract (54, 55), suggesting that metabolites may play a pivotal role in the intricate interplay between gut microbiota and host responses. The relationship between metabolites and cytokines was found to be closer than that with microbiota, indicating that metabolites may exert a more direct influence on pregnancy immunity than gut microbes. In our research, certain microbes may indirectly influence immune function by modulating the upregulation or downregulation of metabolites. For example, OTU_82, which belongs to *Ruminococcus callidus*, may modulate the levels of TNF-α and IL-2 by decreasing the level of deoxycholic acid. *Ruminococcus* participates in the oxidation and epimerization of hydroxyl groups at the C3, C7, and C12 positions in bile acid metabolism (56). A previous study believed that a high level of deoxycholic acid may activate inflammatory (25). An *in vitro* experiment demonstrated that deoxycholic acid promotes the release of TNF-α (57). Our results are consistent with these findings. Regrettably, this study was observational in nature. Although we conducted directional mediation effect analysis to infer the associations among microbiota, metabolism, and immunity, it does not prove a causal relationship between them. The findings still require experimental validation.

There are several limitations in our research. Firstly, the small sample size may limit our ability to effectively account for errors caused by individual differences. Secondly, this study is a cross-sectional study, and due to its inherent limitations, we cannot control for potential influences of confounding factors such as diet. Lastly, despite proposing some hypotheses regarding biological mechanisms through the integration of microbiota, metabolism, and immune data sets, causal relationships cannot be confirmed. The dynamic associations among microbiota, metabolism, and immunity during pregnancy still require validation through multicenter clinical trials with large sample sizes and fundamental experiments. Despite these limitations, this study has offered new insights into our understanding of the connections between the gut microbiome, metabolome, and the immune system during pregnancy.

In summary, our study revealed complicated associations among gut microbiota, metabolites, and immune during pregnancy, and identified some specific metabolites which may act as mediators between symbiotic microorganisms and immune homeostasis. This study provided a comprehensive landscape of the gut microbiota and metabolites in pregnant women and expanded our current knowledge regarding how immune function is affected by gut microbiota and metabolites.

## MATERIALS AND METHODS

### Study design and participants

Thirty pregnant women and 15 non-pregnant women were recruited from the First Affiliated Hospital of Jinan University between February 2019 and August 2020. The inclusion criteria for pregnant participants were as follows: aged between 18 and 34 years, naturally conceived with a singleton pregnancy, and a pre-pregnancy BMI ranging from 18.5 to 21.9 kg/m^2^. Pregnant women with complications such as gestational hypertension, gestational diabetes, or any immune-related disorders were excluded. The control group comprised healthy females aged between 18 and 34 years with a BMI between 18.5 and 21.9 kg/m^2^. All participants reported no use of probiotics or antibiotics in the 6 months prior to participating in the study. Additionally, we also recorded the basic physiological information such as age, weight, and height.

### Sample collection

Fecal and peripheral blood samples from pregnant women were collected during the period from the 37th week of pregnancy until the onset of labor. For the non-pregnant women, samples were collected on the 14th day of their menstrual cycle. Participants defecated onto a sterile pad, after which professionals collected approximately 1 g of feces using a sterile spoon and transferred it into a sterile tube. The fecal samples were transported under cryogenic conditions and stored at −80°C until examined. Additionally, 5 mL of peripheral blood was drawn from fasting participants by a nurse into an EDTA anticoagulant tube. The blood sample was allowed to sit at room temperature for 30 minutes and then centrifuged at 4°C at 2,000 rpm for 10 minutes. The supernatant serum was collected and stored in a refrigerator at −80°C.

### 16S rRNA gene sequencing and species annotation

Total genome DNA of the gut microbiota from samples was extracted by the CTAB method, and then DNA purity and concentration were detected by agarose gel electrophoresis. DNA was placed in a centrifuge tube and diluted to 1 ng/µL with sterile water. The V3 to V4 region of the 16S rRNA gene was amplified using specific primer (341F/806R) with the barcode. Using TruSeq DNA PCR-Free Sample Preparation Kit (Illumina, USA) generated sequencing libraries. The library was sequenced on an Illumina NovaSeq platform. Raw tags were truncated and chimera-filtered according to the QIIME (V1.9.1, http://qiime.org/scripts/split_libraries_fastq.html) quality-controlled process (58). Ultimately, sequences with ≥97% similarity were assigned to the same OTUs using the Uparse software (Uparse v7.0.1001, http://www.drive5.com/uparse/) (59). Representative sequences for each OTU were screened for further annotation.

### Microbial analysis

To identify representative microbes for the two groups, we conducted random forest analysis using R package “randomForest” in RStudio software (version 1.4.1717). Subsequently, the top 100 OTUs of variable importance predicted by random forest were clustered using the Ward clustering algorithm via the R package “WGCNA.” The microbes contained in the gray module are microbes that do not belong to any other modules, and they were excluded from subsequent analysis. The average abundance of modules between the two groups was compared using the Wilcoxon rank-sum test method.

### Untargeted LC-MS-based metabolomics

Fecal samples and plasma samples were detected by liquid chromatography-tandem mass spectrometry (LC-MS/MS) analysis. The samples were thawed at 4°C and mixed with cold methanol/acetonitrile (2:2:1, vol/vol). The mixture was centrifuged for 15 minutes (14,000 × *g*, 4°C). The supernatant was dried in a vacuum centrifuge. For LC-MS analysis, the samples were re-dissolved in 100 µL acetonitrile/water (1:1, vol/vol) solvent. To monitor the stability and repeatability of instrument analysis, quality control (QC) samples were prepared by pooling 10 µL of each sample and analyzed together with the other samples. The QC samples were inserted regularly and analyzed in every five samples.

Analyses were performed using a ultra-high performance liquid chromatography (UHPLC) (1290 Infinity LC, Agilent Technologies) coupled to a quadrupole time-of-flight (AB Sciex TripleTOF 6600) in Shanghai Applied Protein Technology Co., Ltd. The metabolites in the samples were detected by UHPLC-Q-TOF MS. The raw data were converted into .mzxml format by ProteoWizard. XCMS software was used to correct the retention time of peak alignment and extract the peak area. Firstly, the data of metabolite structure identification were preprocessed for the extracted data, then the quality of the experimental data was evaluated and analyzed.

### Metabolomics data analysis

Metabolomic data analysis was performed using the APT-BioCloud platform (http://cloud.aptbiotech.com/#/login). The OPLS-DA was used for multivariate data analysis. Additionally, the robustness of this model was evaluated by the sevenfold cross-validation and response permutation testing. The VIP value of each metabolite in the OPLS-DA model was calculated to indicate its contribution to the classification. Metabolites with VIP value >1 and *P* values <0.05 of Student’s *t*-test were considered statistically significant. Differentially metabolites were further mapped to the KEGG database to identify the altered biological pathways.

### Detection of plasma cytokines

Concentrations of IFN-γ, TNF-α, interleukins IL-1β, IL-2, IL-4, IL-6, IL-10, and IL-12 were measured by multiplex bead assay analysis according to the manufacturer’s instructions. Differences of cytokines between groups were calculated and visualized using GraphPad Prism 8.0.2 software.

### Multi-omics analysis

We conducted correlation analysis of gut microbiota, metabolites, and cytokines based on the Spearman method. In addition, we also performed a directional mediation analysis to evaluate the potential mediation by metabolites. We calculated the mediation effect by the R package “bruceR.” To test the mediation effect, a bootstrap method was conducted with 1,000 bootstrap samples. The Sankey diagram was created to visualize microbes affect cytokines by metabolites and drawn using the R package “ggalluvial.”

### Statistical analysis

The measurement data conforming to the normal distribution were expressed by mean ± standard deviation, and Student’s *t*-test was used to evaluate differences between the two groups. The nonparametric Mann-Whitney test was conducted for data that did not conform to a normal distribution. Statistical analysis was performed using RStudio software (version 1.4.1717) or GraphPad Prism software (version 8.0.2).

## Data Availability

Raw sequencing data have been publicly deposited and are available at the NCBI Sequence Read Archive, with BioProject accession no. PRJNA975340. The metabolomics data for the participants and the STORMS checklist are available in the Mendeley Data Repository (https://data.mendeley.com/datasets/t4gw54s8k3/2).

## SUPPLEMENTAL MATERIAL

Supplemental Figures msystems.01252-23-s0001.pdf Figures S1 to S6.

Supplemental tables msystems.01252-23-s0002.xlsx Tables S1 to S4.

ASM does not own the copyrights to Supplemental Material that may be linked to, or accessed through, an article. The authors have granted ASM a non-exclusive, world-wide license to publish the Supplemental Material files. Please contact the corresponding author directly for reuse.
