# Supplementary material for: Multi-omics analysis reveals the associations between altered gut microbiota, metabolites, and cytokines during pregnancy
Source: mSystems. 2024 Feb 7;9(3):e01252-23. doi: 10.1128/msystems.01252-23 (PMC10949498; doi:10.1128/msystems.01252-23)

Supplementary Figure S1 Top 100 microbial biomarkers based on random forest model. Features are ranked by their contributions to classification accuracy (Mean Decrease Accuracy).

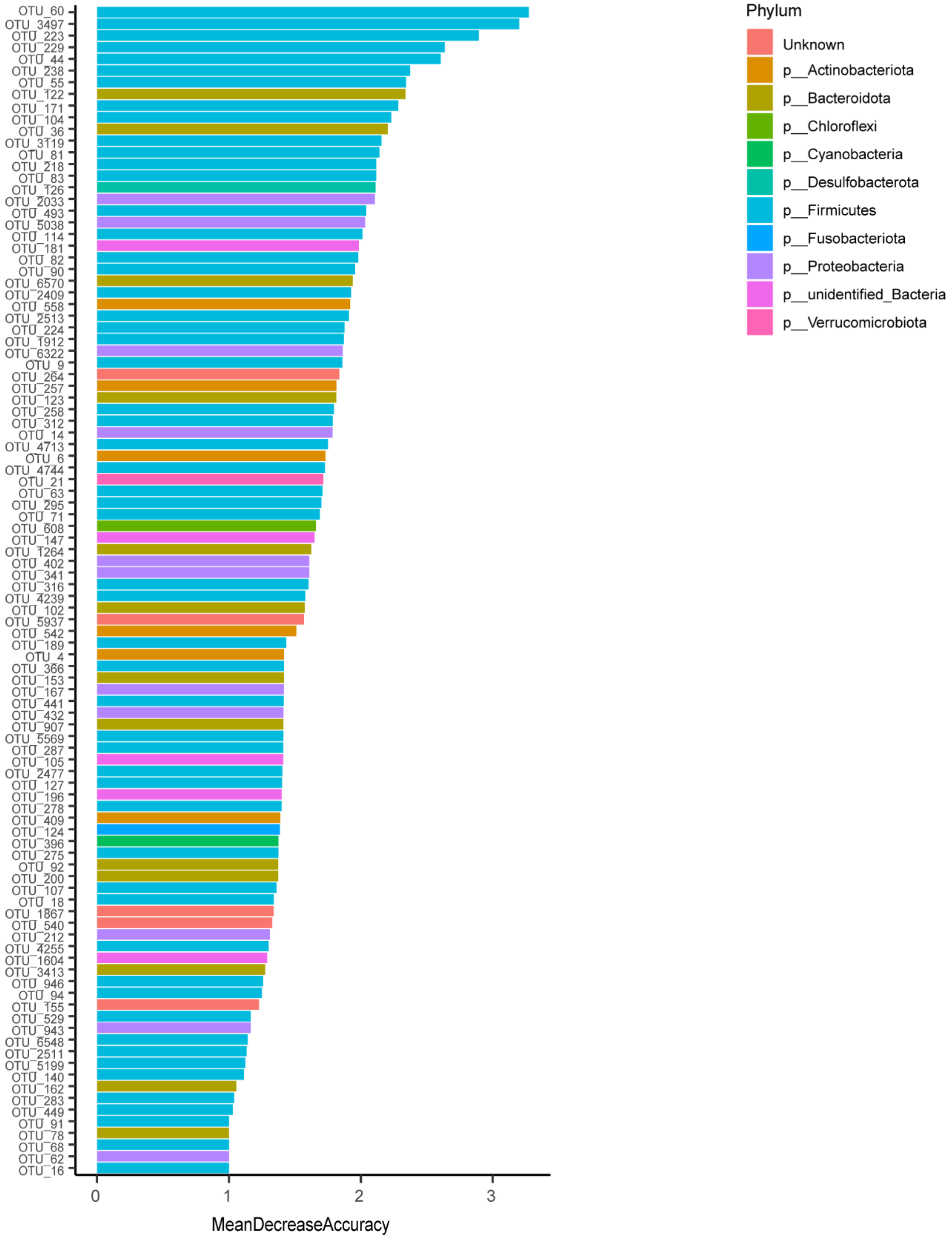

Supplementary Figure S2 Comparative analysis of relative abundance for OTU_4, OTU_6, OTU_16, and OTU_82 between the pregnancy and control groups. Differences between groups were calculated using the Wilcoxon rank-sum test. *, $P < 0.05$. **, $P < 0.01$. ***, $P < 0.001$.

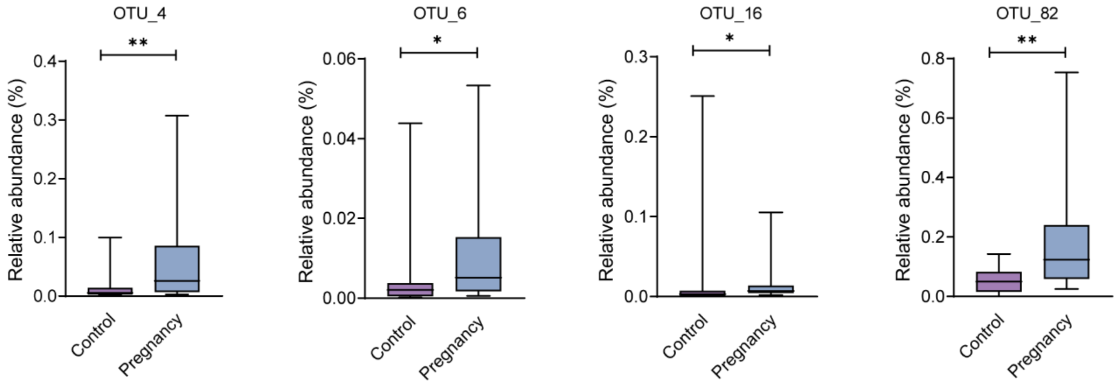

Supplementary Figure S3 OPLS-DA score plot of plasma samples from pregnancy group and control group in positive ion model ES (+) and negative ion model ES (-), respectively. Samples from the same group are represented by points of the same color. The distribution of points reflects differences between and within groups.

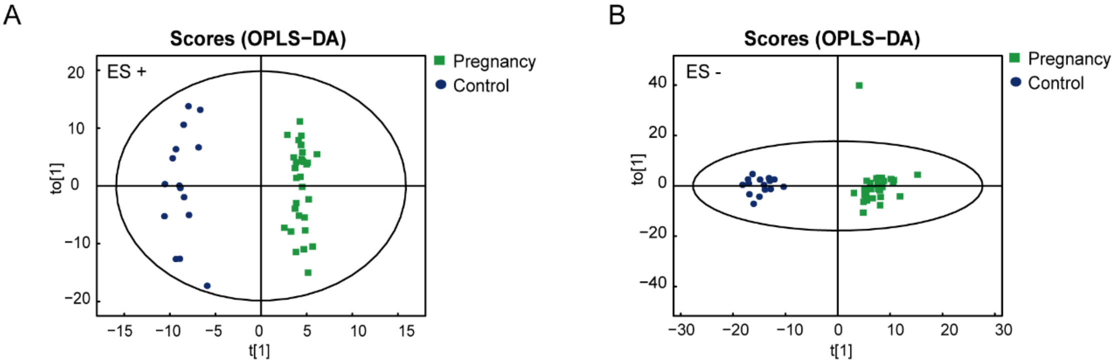

Supplementary Figure S4 The permutation test of OLPS-DA model of fecal metabolites in (A) positive ion mode and (B) negative ion mode. The permutation test of OLPS-DA model of plasma metabolites in (C) positive ion mode and (D) negative ion mode. The X-axis represents permutation retention percentage, while the Y-axis represents the values of R^2 and Q^2. Green points correspond to R^2 values, and blue points correspond to Q^2 values, with two dashed lines indicating the regression lines for R^2 and Q^2. In the top-right corner, R^2 and Q^2 values are presented for permutation retention equal to 1, representing the original model's R^2 and Q^2 values. As permutation retention gradually decreases, both R^2 and Q^2 values for the random models decrease progressively, indicating the absence of overfitting in the original model and its robustness.

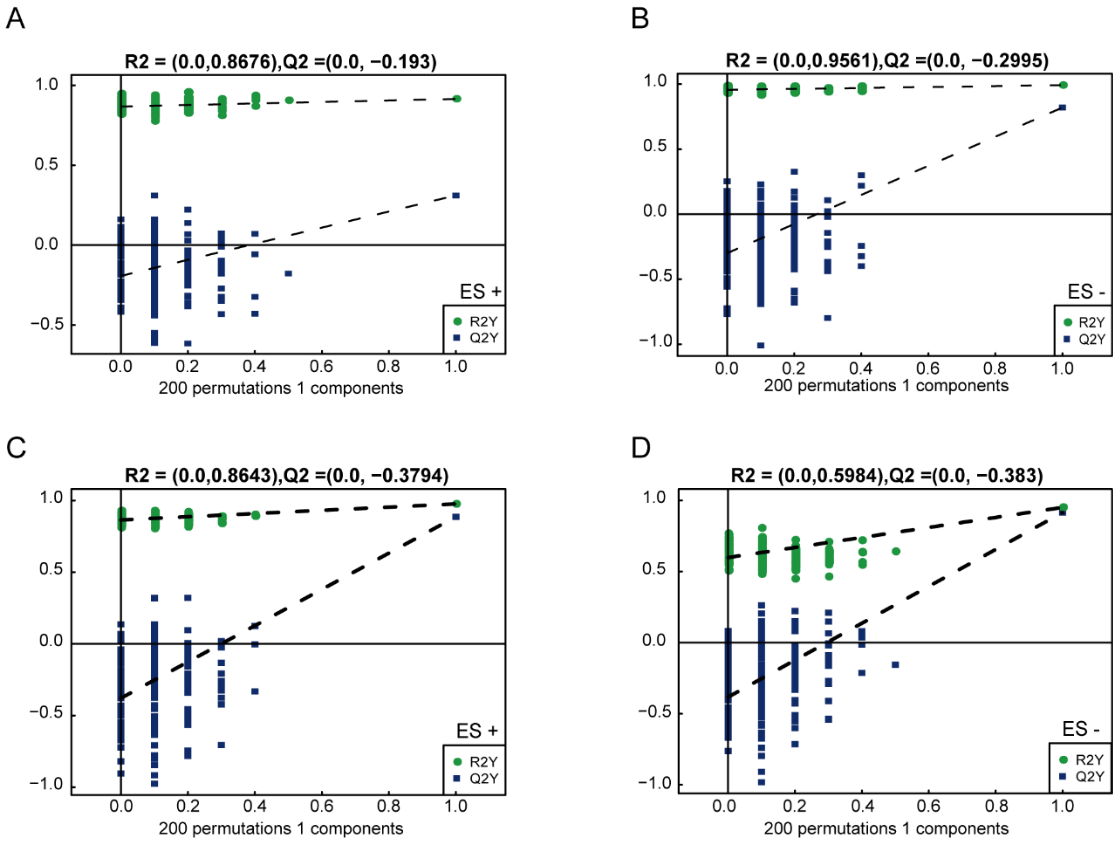

Supplementary Figure S5 The metabolites shared by plasma and feces were different in plasma between pregnancy group and control group. (A) Metabolites in positive ion mode. (B) Metabolites in negative ion mode. Differences between groups were calculated using the Wilcoxon rank-sum test. *, $P < 0.05$. **, $P < 0.01$. ***, $P < 0.001$.

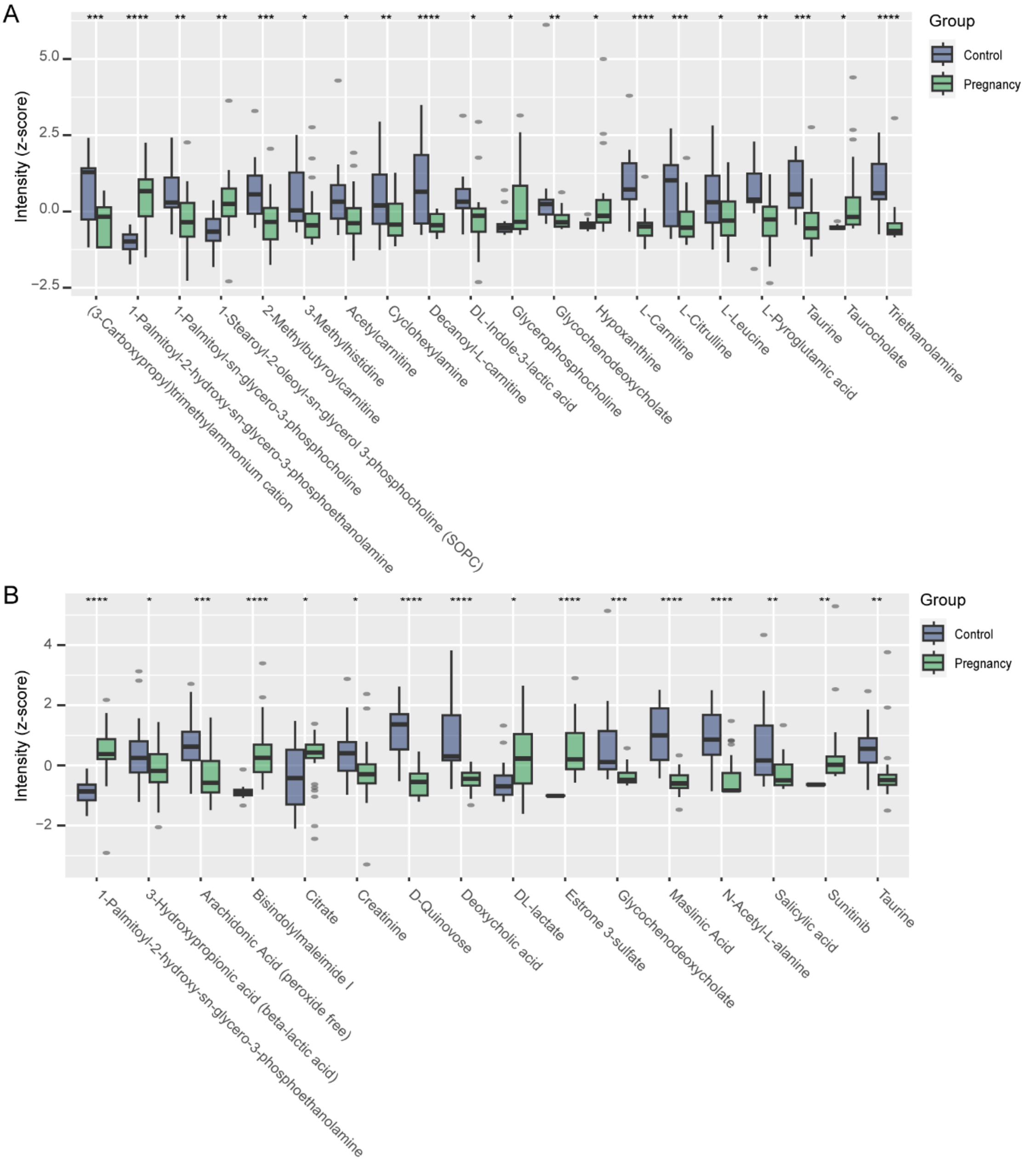

Supplementary Figure S6 (A) Relative abundance of OTU_82 in the control and pregnancy groups. (B) Peak intensity of deoxycholic acid in the control and pregnancy groups. (C) Relative abundance of OTU_943 in the control and pregnancy groups. (D) Peak intensity of Arachidonic acid in the control and pregnancy groups. Differences between groups were calculated using the Wilcoxon rank-sum test. *, $P < 0.05$. **, $P < 0.01$. ***, $P < 0.001$.

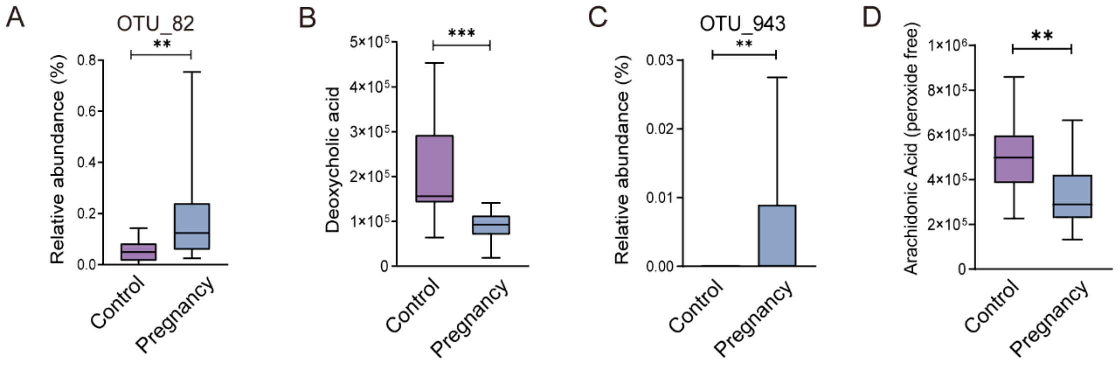

Supplement: Supplemental Figures — Figures S1 to S6. [file msystems.01252-23-s0001.pdf]
